# Safety of coagulation factor concentrates guided by ROTEM™-analyses in liver transplantation: results from 372 procedures

**DOI:** 10.1186/s12871-019-0767-x

**Published:** 2019-06-11

**Authors:** Matthias Hartmann, Caroline Walde, Daniel Dirkmann, Fuat H. Saner

**Affiliations:** 1 Klinik für Anästhesiologie und Intensivmedizin, Universitätsklinikum Essen, Universität Duisburg-Essen, Hufelandstr. 55, 45122 Essen, Germany; 2 Klinik für Allgemein-, Viszeral- und Transplantationschirurgie, Universitätsklinikum Essen, Universität Duisburg-Essen, 45122 Essen, Germany

**Keywords:** Liver transplantation, Haemostasis, Fibrinogen, Prothrombin complex concentrates, Tranexamic acid

## Abstract

**Background:**

Most centres use fresh frozen plasma (FFP) based protocols to prevent or treat haemostatic disturbances during liver transplantation. In the present study, we used a rotational thrombelastometry (ROTEM™, TEM, Munich, Germany) guided haemostasis management with fibrinogen concentrates, prothrombin complex concentrates (PCC), platelet concentrates and tranexamic acid without FFP usage and determined the effect on 30 day mortality.

**Methods:**

Retrospective data analysis with 372 consecutive adult liver transplant patients performed between 2007 and 2011.

**Results:**

Thrombelastometry guided coagulation management resulted in a transfusion rate for fibrinogen concentrates in 50.2%, PCC in 18.8%, platelet concentrates in 21.2%, tranexamic acid in 4.5%, and red blood cell concentrates in 59.4%. 30 day mortality for the whole cohort was 14.2%. The univariate analyses indicated that nonsurvivors received significantly more fibrinogen concentrates, PCC, red blood cell concentrates, platelet concentrates, and infusion volume, and had a higher MELD score. However, association with mortality was weak as evidenced by receiver operating characteristic curve analyses. Further univariate analyses demonstrated, that up to 8 g of fibrinogen did not increase mortality compared to patients not receiving the coagulation factor. Multivariate analysis demonstrated that platelet concentrates (*p* = 0.0002, OR 1.87 per unit), infused volume (*p* = 0.0004, OR = 1.13 per litre), and MELD score (*p* = 0.024; OR 1.039) are independent predictors for mortality. Fibrinogen concentrates, PCC, and red blood cell concentrates were ruled out as independent risk factors.

**Conclusions:**

ROTEM™ guided substitution with fibrinogen concentrates and PCC does not negatively affect mortality after liver transplantation, while the well-known deleterious effect associated with platelet concentrates was confirmed.

## Background

During liver transplantation, relevant disturbances of haemostasis are common and its pathophysiology is complex [1]. Accordingly, precise and timely monitoring to guide coagulation management is desirable. Both conventional and bedside methods are frequently used [2]. While the usefulness of conventional laboratory methods is largely hampered by long turnaround times, bedside monitoring allows for the rapid and comprehensive diagnosis of coagulopathies but requires additional technical skills of the anaesthetist [3, 4]. Viscoelastic methods like thromboelastography and rotational thromboelastometry (ROTEM™) are the most commonly used point-of-care methods during liver transplantation [2]. Rotational thromboelastometry (ROTEM™) is capable to measure the clot firmness in whole blood samples in a time dependent fashion [5, 6]. The use of various assays performed in parallel allows to distinguish the underlying mechanisms for coagulopathies [7].

As the reason for coagulopathy can be determined by use of ROTEM™, a targeted therapy with coagulation factor concentrates, platelets, and fibrinolysis inhibitors without the use of fresh frozen plasma and the prophylactic use of antifibrinolytics is increasingly used. For this purpose, a ROTEM™ based algorithm has been proposed [8].

The use of fresh frozen plasma is hampered by the fact, that there are important data on its risks available, but only sparse evidence for its efficacy [9, 10]. In contrast, cautious recommendations have been made regarding the use of fibrinogen concentrates in bleeding patients, and available pharmacovigilance data suggest the safety of PCC [10–12].

In the present retrospective study, we investigated the influence of the ROTEM™ guided use of fibrinogen concentrates, PCC, platelet concentrates, and tranexamic acid on 30 day mortality in liver transplant patients.

## Methods

### Patient data

After approval by the local ethics committee, data from 457 consecutive adult liver transplantation procedures performed between 2007 and 2011 were retrospectively analysed. 144 patients with a body weight of less than 30 kg were excluded to avoid heterogeneity due to the inclusion of pediatric liver transplantations. Moreover, 18 patients getting fresh frozen plasma or incomplete data were excluded.

### Procedure

All liver transplantations were performed with organs from deceased donors. Surgery was performed through a star incision in the upper abdomen with a vena cava replacement technique. None of the transplanted patients underwent a venovenous bypass.

For the induction of anaesthesia, thiopental was used. Isoflurane and fentanyl were used for maintenance of anaesthesia. Endotracheal intubation and surgery were facilitated by rocuronium. For the haemodynamic monitoring, a radial artery catheter, a central venous catheter, a pulmonary artery catheter as well as transoesophageal echocardiography were used. The femoral vein pressure was monitored for the detection of possible caval vein stenosis. For the treatment of hypovolemia and anaemia, a rapid infusion device (FMS-2000, Belmont Instruments Corporation, Billerica, MA, USA), connected to a large bore dialysis catheter and filled with normal saline (0.9%) and red blood cell concentrates were used as required. Intra-operative cell-salvage was used in all patients without cancer, retranfusion required the sampling of at least 300 ml. Patients received 5000 U heparin per 24 hours during their stay on the intensive care ward.

### Evaluation of haemostasis

For the bed side evaluation of haemostasis, ROTEM™ devices and a coulter counter were used. Moreover, the conventional laboratory assays international normalized ration (INR), activated partial thromboplastin time (aPTT), fibrinogen concentration, antithrombin, platelet count, and hemoglobin concentration (Hb) were measured but not used for therapeutic decisions due to long turnaround times.

The ROTEM™ device measures the time dependent development of clot firmness of a whole blood sample. Thus, both the involvement of coagulation factors and platelets can be investigated. There are four important variables obtained from the thromboelastogram [7, 13]. The clotting time defined as the time from recalcification and activation of the samples to clot formation is prolonged in patients with coagulation deficiencies, heparin therapy, or on oral anticoagulation. Clot formation time and angle alpha describe the kinetics of clot formation. Maximum clot firmness is affected by fibrinogen levels and platelet count. Four tests were used in the present study. EXTEM™ activates coagulation by the addition of tissue factor and has similarities to the INR. INTEM™ is activated by elagic acid, which is an activator of the intrinsic system similar to laboratory aPTT. FIBTEM™ is an assay activated by tissue factor in the presence of a platelet inhibitor (cytochalasin D) and maximum clot firmness is therefore a specific measure of fibrinogen concentration. APTEM™ is a tissue factor activated assay combined with a fibrinolysis inhibitor (aprotinin). Hyperfibrinolysis can be diagnosed by comparison of EXTEM™ and APTEM™ curves. Measurements of haemostasis were routinely performed at the beginning of surgery, during the anhepatic phase, subsequent to liver graft reperfusion, and at the end of the procedure. When haemostatic derangements occurred, further measurements were performed according to the discretion of the anaesthetist.

Haemostatic interventions with fibrinogen concentrate and PCC (CSL-Behring, Marburg, Germany), platelet concentrates, and tranexamic acid were based on the observation of diffuse bleeding and the results of ROTEM™ analyses according to a recently published algorithm [8]. Composition of the four factor PCC preparation used (CSL-Behring, KCentra 500 units) was as follows (see prescribing information): factor II (380–800 units), factor VII (200–500 units), factor IX (400–620 units), factor X (500–1020 units), protein C (420–820 units), protein S (240–680 units), heparin (8–40 units), antithrombin (4–30 units).

### Data analysis

Data on patients´ age, sex, body mass index, MELD score, and donor risk index as well as the diseases necessitating liver transplantation were recorded. Besides mortality, the use of fibrinogen concentrates, PCC, platelet concentrates, red blood cells, and tranexamic acid, as well as laboratory haemostasis findings (aPTT, INR, platelet count) and bed side findings (ROTEM™) were registered. Moreover, the infusion volume via the rapid infusion system as well as the autotransfusion volume obtained from the cell salvage device, when applicable, are given. For the evaluation of red blood cell mass loss a recently validated algorithm was used [13]. In short, the following equation was used: Lost RCM (mL) = patient’s estimated blood volume (mL) x (preoperative hematocrit in % - postoperative haematocrit in %) + transfused leukocyte-depleted red blood cell in units × 213 × 70% + transfused cell saver blood in mL × 55%. Blood volume was calculated from the body weight (75 ml/ kg in men, and 65 ml/ kg in women) [14].

### Statistics

The frequencies and the respective amount of blood product used were evaluated. For statistical evaluation, SPSS (version 24, IBM, USA) was used. For the description of results both mean and standard deviation and median and percentiles are given. For statistical evaluation we used tests which are not dependent on normal distribution. To determine a potential association of blood product usage with outcome, both univariate and multivariate analyses were used. For univariate analyses, the Wilcoxon test (unpaired or paired) or Chi-square test as well as receiver operating characteristic curves and the asymptotic significance were used. Multivariate analyses were performed using binary logistic regression using the backward likelihood ratio method. Probability of score statistic for variable entry was 0.05, probability of LR statistic to remove a variable was 0.1, the numbers of iterations was limited to 20, and the cut-off value for classification was 0.5.

## Results

### Use of blood products

The Mortality of the 372 transplanted patients (230 male, 142 female) was 14.2 %. Data on age, sex, body mass index, MELD score, donor risk index as well as the diseases leading to liver transplantation are given in Tables 1 and 2, respectively.

**Table 1:** Patients and donor characteristics given as mean and standard deviation (SD) as well as median and range

	mean ± SD	median [range]
age (years)	51 ± 12	53 [10–74]
MELD	20 ± 9.6	18 [6–40]
donor risk index	1.74 ± 0.36	1.76 [1.0–2.26]
body mass index	26.6 ± 4.9	26 [15–48]

**Table 2:** Diseases finally resulting in liver transplantation. Note, that hepatocellular carcinoma were associated with hepatitis B and C in 25 cases

disease	number of cases
alcohol	80
hepatocellular carcinoma	77
hepatitis B and C	62
cholestatic liver diseases	40
inherited liver diseases	26
NASH	23
retransplantation	15
acute liver failure	10
autoimmune liver disease	10
hemochromatosis	7
drug toxicity	5
tumor (not HCC)	5
others	12

**Fig. 1:**
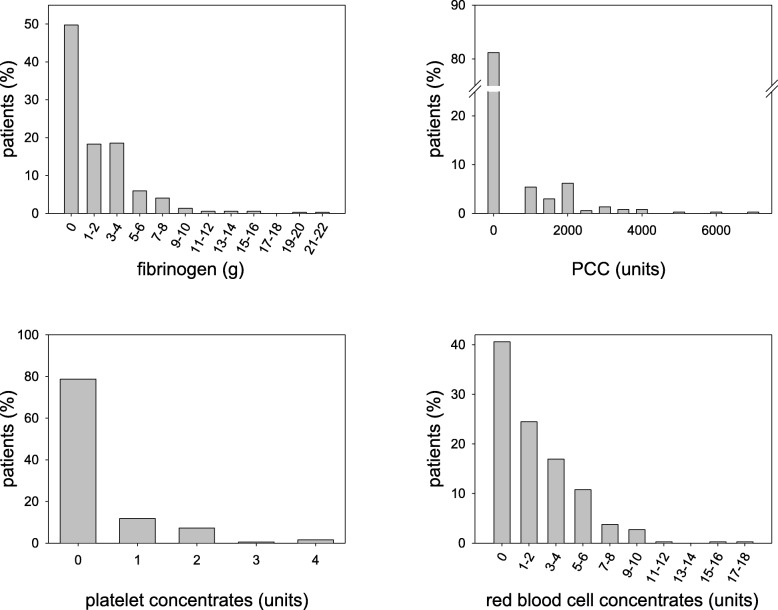
Frequencies of intraoperative blood product use in 372 patients undergoing liver transplantation. Fibrinogen concentrates, PCC, platelet concentrates and red blood cell concentrates used were grouped and the number of patients is presented. Notably, blood products were not transfused in many cases. (PCC: prothrombin complex concentrate)

In 372 liver transplantation procedures, administration of blood products was as follows: Fibrinogen concentrate was administered in 187 patients (range 1–22 g), 70 patients received four factor PCC (range 1000-7000 U), 79 patients received platelet concentrates (range 1–4 units), and 221 patients received red blood cell concentrates (range 1–17 units). The details of blood products administered are provided in Fig. 1.

Anemia at the begin of surgery was associated with transfusion of red blood cell concentrates, linear regression analysis demonstrated that a lowered hemoglobin concentration by 1 g/dl increased transfusion of red blood cell concentrates by 0.68 units (*r* = 0.44). Hypofibrinogenemia was associated with increased fibrinogen concentrate substitution: a decrease of fibrinogen by 100 mg/dl was associated with the substitution of 0.88 g fibrinogen (*r* = 0.36).

### Association of blood product use and mortality: univariate analysis

**Fig. 2:**
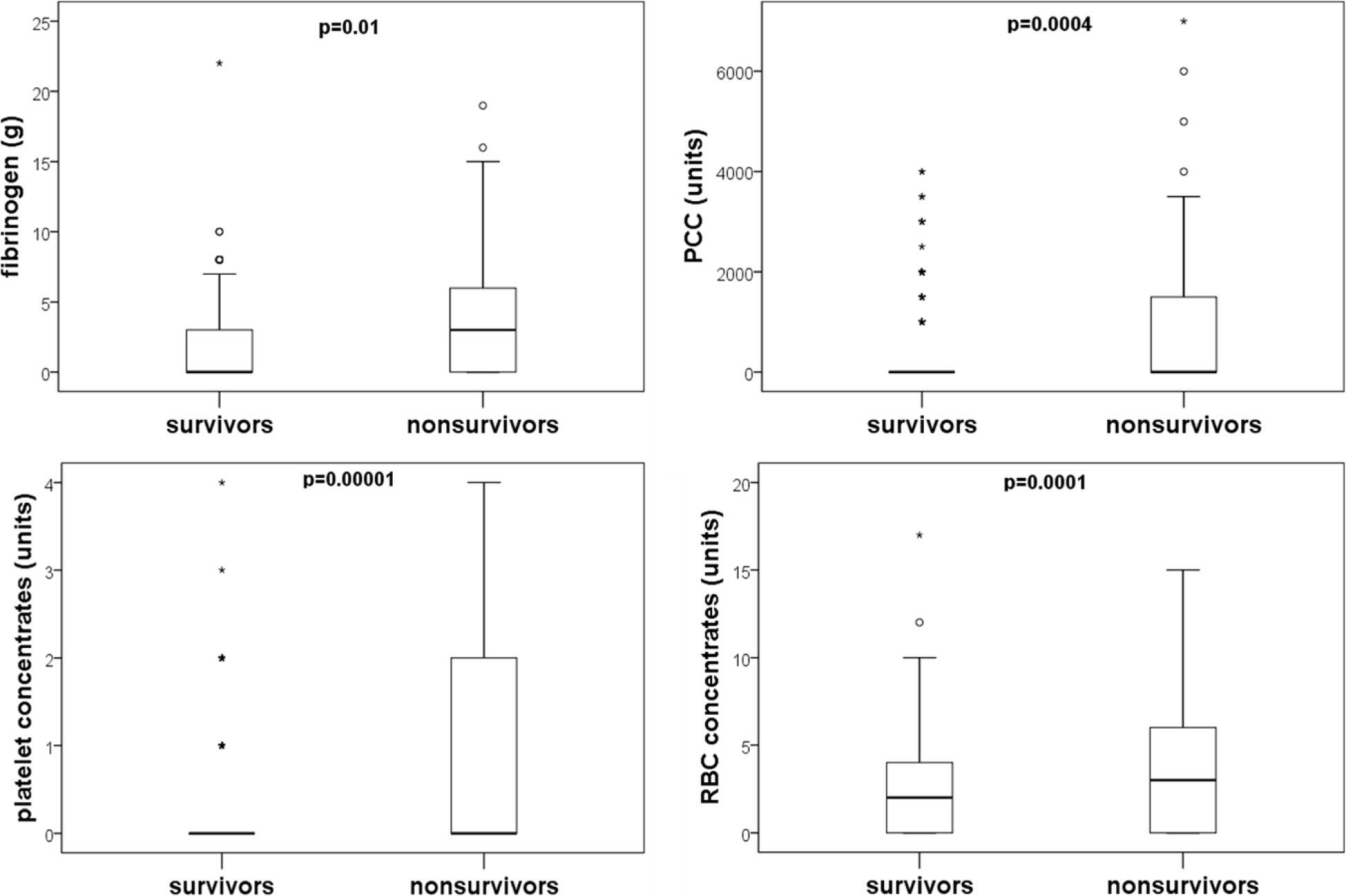
Blood product use in survivors and nonsurvivors of orthotopic liver transplantation. Nonsurvivors were more likely to receive fibrinogen concentrates, PCC, platelet concentrates, and red blood cell concentrates. Differences between groups were evaluated using the unpaired Wilcoxon test. (PCC: prothrombin complex concentrate; RBC: red blood cell concentrate)

In order to obtain a first insight into the potential association of fibrinogen concentrate, PCC, platelet concentrates, and red blood cell concentrates with 30 day mortality, box plots are shown in Fig. 2. In comparison to survivors, the amount of blood products was significantly higher in nonsurvivors. Mean value and standard deviation of the respective blood product was 1.8 g ± 2.6 g fibrinogen in survivors versus 4.25 g ± 4.98 g in nonsurvivors, 299 units ±761 units versus 896 units ±1603 units PCC, 2.1 units ±2.4 units versus 4.0 units ±3.6 units red blood cell concentrates, and 0.25 units ±0.599 units versus 0.91 units ±1.3 units platelet concentrates.

**Fig. 3:**
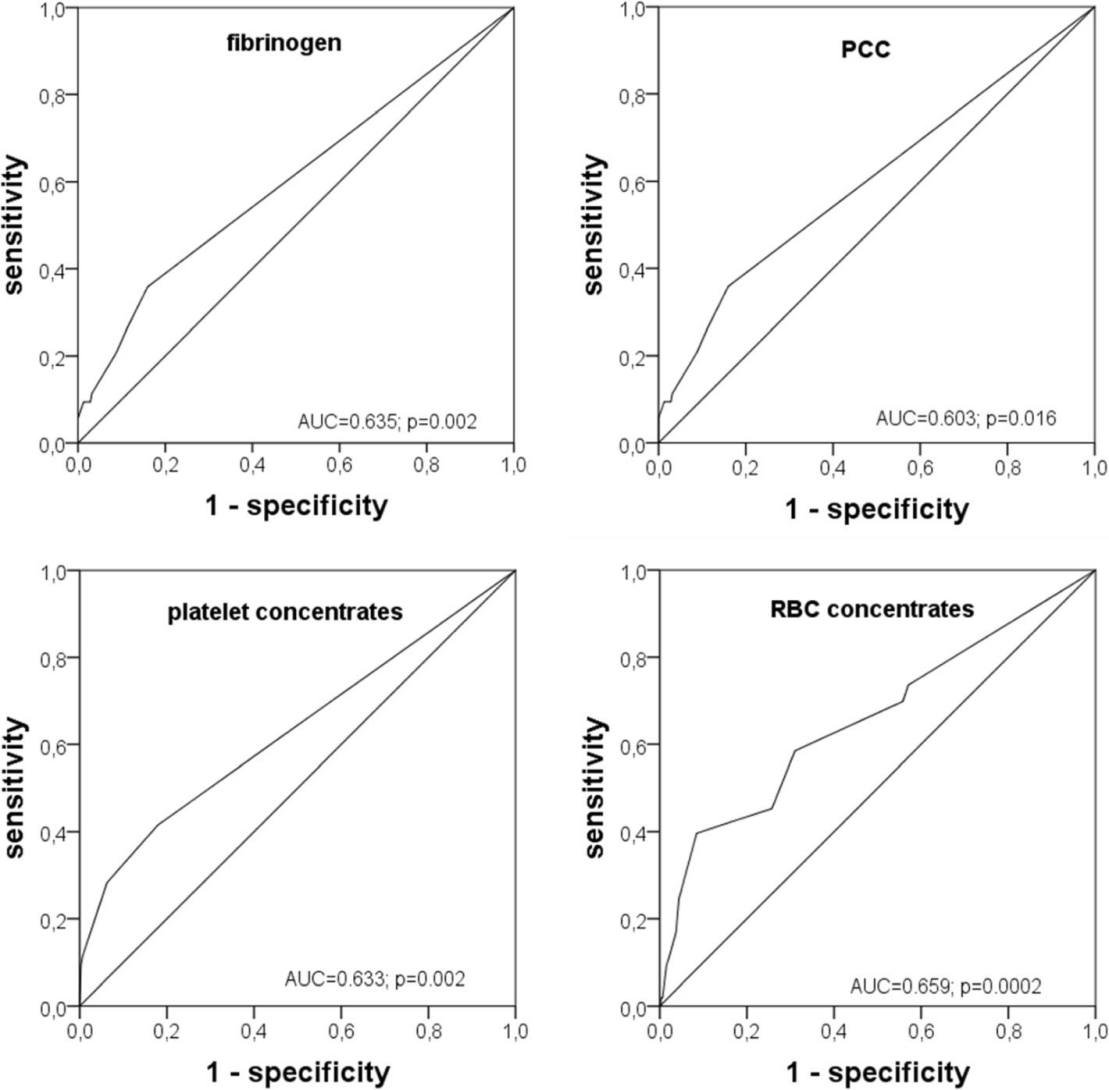
Receiver operating characteristic curves demonstrating the association between the blood products and 30 day mortality. Fibrinogen, PCC, platelet concentrates, and red blood cell concentrates were associated with mortality as evidenced by asymptotic significance. Areas under curves (AUC), however, demonstrated that the association was low

To further characterize the association of blood products with outcome, receiver operating characteristic curves and the corresponding areas under the curves were evaluated. The results, shown in Fig. 3, demonstrate an only weak association of the respective blood product use with survival (AUC between 0.603 and 0.659).

### Association of red blood cell mass loss and mortality: univariate analysis

The median and range of red blood cell mass loss was 424 ml [− 479 ml - 2296 ml]. Red blood mass loss was 398 ml [− 479 ml - 2296 ml] in survivors and 801 ml [− 26 ml - 2149 ml] in nonsurvivors (*p* < 0.001). Area under curve of the receiver operating characteristic curve was 0.678 (*p* = 0.000062). Linear regression demonstrated that red blood cell mass loss was associated with transfusion of red blood cell concentrates (*r* = 0.69), fibrinogen (*r* = 0.54), and platelet concentrates (*r* = 0.49).

### Mortality and amount of fibrinogen concentrates transfused

**Fig. 4:**
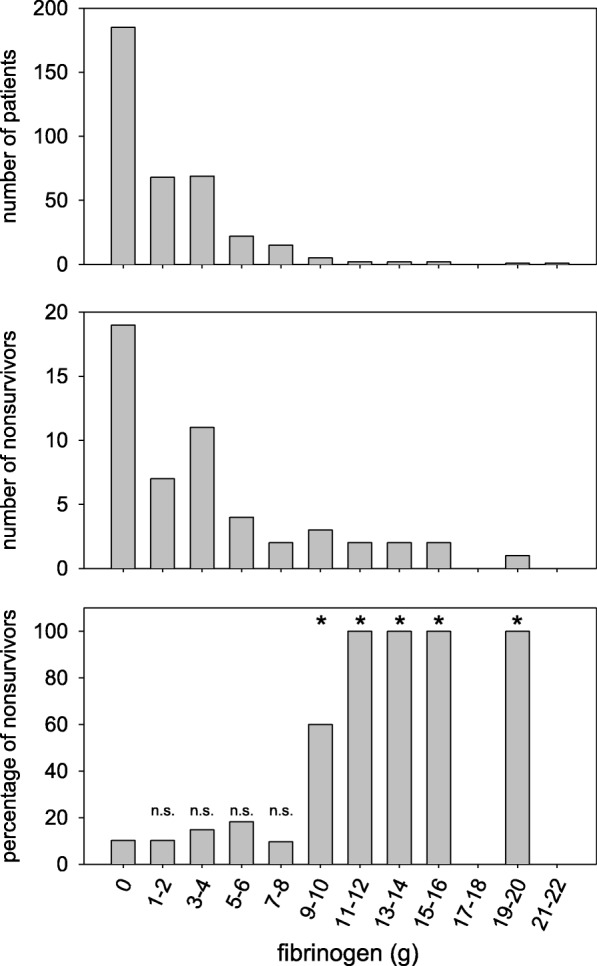
Effect of the fibrinogen dosage on nonsurvival. Fibrinogen doses were grouped and eventual differences in nonsurvival were evaluated. No increase in 30 day mortality was seen in when 1 to 8 g of fibrinogen concentrate was given. For statistical evaluation the Chi square test was used. n.s.: non significant difference between 0 g fibrinogen and the respective group; *: significance *p* < 0.05

The most common blood product used in the present cohort was fibrinogen, permitting a detailed analysis of eventual mortality associated with fibrinogen concentrate use. Therefore, patients were grouped according to the amount of fibrinogen concentrate applied during surgery (0 g, 1–2 g, 3–4 g, 5–6 g, 7–8 g, 9–10 g, 11–12 g, 13–14 g, 15–16 g, 17–18 g, 19–20 g). As shown in Fig. 4, 185 out of 372 patients were not substituted with fibrinogen. In 174 out of 372 patients, fibrinogen was applied in a range from 1 g to 8 g. Comparison of mortality in the different groups in comparison to patients not substituted with fibrinogen demonstrated excluded a significant increase up to 8 g fibrinogen. In those few fibrinogen substituted patients (13 out of 187 patients) receiving more than 8 g fibrinogen, 10 patients died. Review of the patient records, however, demonstrated that the death of only one patient was attributable to a thrombotic event (myocardial infarction). Further analyses on the potential impact of fibrinogen concentrates are shown in the multivariate analyses section.

### Association of infused volume, cell salvage autotransfusion, tranexamic acid and MELD score with mortality: univariate analyses

Blood volume replacement was conducted via a rapid infusion system: both normal saline (0.9%) as well as red blood cell concentrates were administered with this device. Median and range of the total infused volume was 7.5 l [1 l - 28.6 l]. Autotransfusion in patients without cancer diagnosis was 379 ml [0 ml - 2967 ml] (median and range). In 62 patients, blood loss was too low for processing with the cell salvage device. Tranexamic acid was used in 91 out of 372 patients, when relevant hyperfibrinolysis was diagnosed. The most common dose was 2 g, followed by 1 g in 11 cases, and 4 g (2 g, once repeated during surgery) in 1 patient. Median MELD score and range were 18 [6–40].

**Fig. 5:**
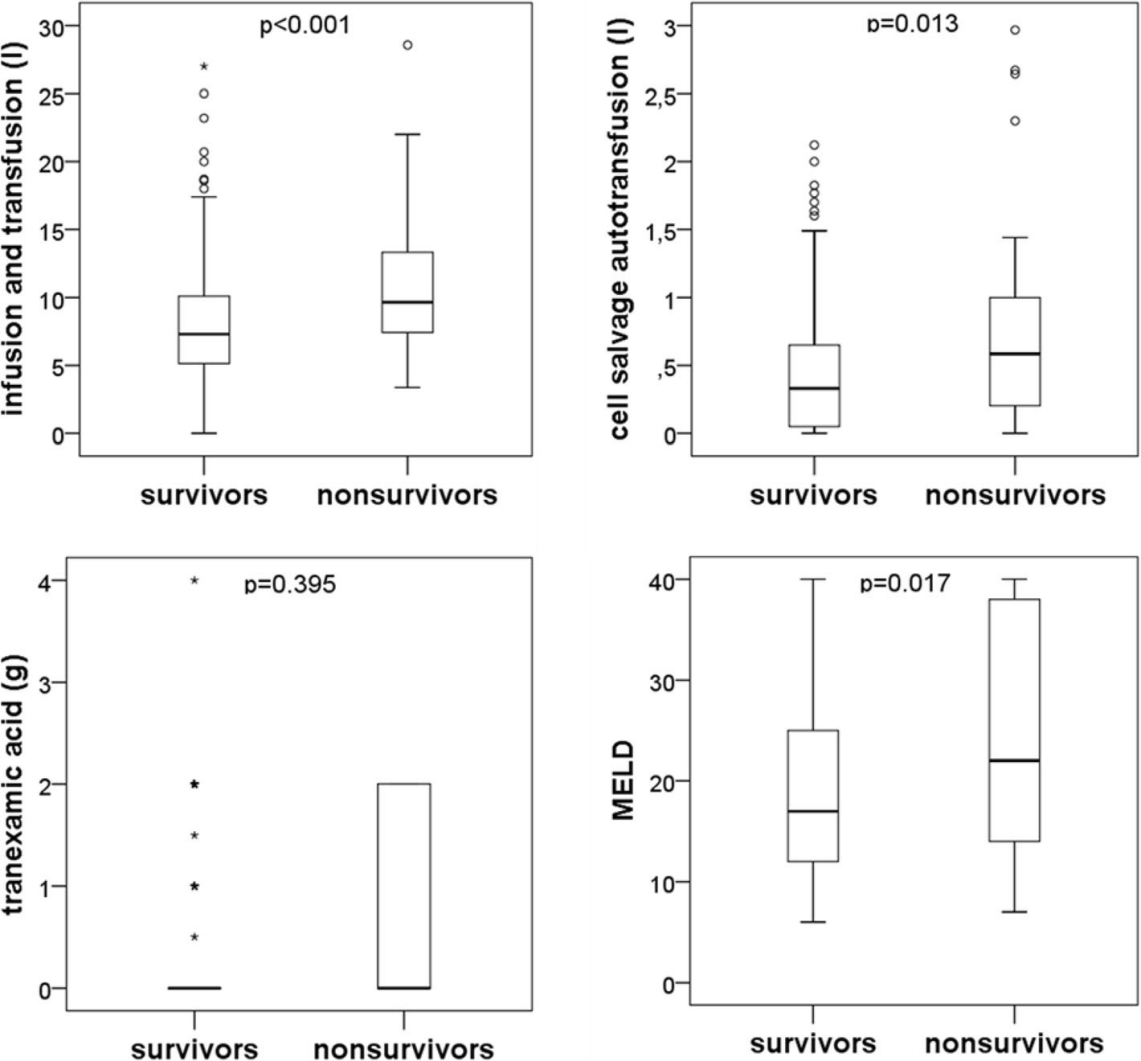
Blood loss (as estimated by volume infused via the rapid infusion system and cell salvage autotransfusion volume, respectively), tranexamic acid usage, and MELD score in survivors and nonsurvivors of liver transplantation. For statistical evaluation, the unpaired Wilcoxon test was used

Infused volume, autotransfusion volume, and MELD score but not tranexamic acid were associated with outcome (Fig. 5). Median infused volume was 7.3 l [0 l − 27 l] in survivors, and 9.7 l [3.4 l – 28.6 l] in nonsurvivors, respectively (*p* < 0.001). Similarly, median autotransfusion volume was 0.33 l [0 l - 2.12 l] in survivors, and 0.59 l [0 l – 2.96 l] in nonsurvivors, respectively (*p* = 0.013). The median MELD score was 17 (range 6–40) in survivors, and 22 (7–40) in nonsurvivors (*p* = 0.017), respectively.

Area under the curve of the receiver operating characteristic curves were 0.697 for infused volume (*p* = 0.00002), 0.643 for autotransfusion (*p* = 0.002), 0.552 for tranexamic acid (*p* = 0.261), and 0.609 for the MELD-score (*p* = 0.017), thus demonstrating that the association of the variables with mortality was fair, at best. It is important to state, that the analysis of autotransfusion was restricted to those patients undergoing cell salvage (*n* = 270).

### Multivariate analysis of the association of coagulation factors and blood products transfused, blood loss, tranexamic acid usage, and MELD-score

To further investigate the association of blood products use with mortality, binary logistic regression analysis was used. Fibrinogen concentrate, PCC, red blood cell concentrates, platelet concentrates, infused and transfused volume as a surrogate parameter of blood loss, tranexamic acid use, and MELD score were included in the analysis. Platelet transfusion rate was the strongest independent predictor for mortality with an odds ratio of 1.87 per unit (*p* = 0.0002). Further variables associated with mortality were blood loss as estimated by the infused volume (OR 1.13 per l; *p* = 0.0004) and the MELD score (OR 1.039; *p* = 0.024). In contrast, fibrinogen concentrates (*p* = 0.90), PCC (*p* = 0.26), red blood cell concentrates (*p* = 0.71), and tranexamic acid (*p* = 0.71) were not associated with mortality in the multivariate analysis. In order to exclude a collinearity between red blood cell concentrates and transfused volume, the multivariate analysis was also performed with an exclusion of red blood cell concentrates with identical findings (data not shown).

### Initial and final ROTEM findings and conventional laboratory findings

Substitution of RBCs, coagulation factors, and platelet concentrates was guided by ROTEM analysis, and the findings at the beginning and end of the transplantation procedure is shown in Table 3. Initial INR was significantly different in survivors and non survivors (1.63 ± 0.62 vs. 1.83 ± 0.68 (*p* = 0.049), while all other ROTEM findings at the begin of the procedure and conventional laboratory findings were not different in survivors and nonsurvivors (data not shown). Median fibrinogen levels moderately decreased during the transplantation as evidenced by both fibrinogen concentration and FIBTEM clot firmness. Similarly, INR increased, which is mirrored by a corresponding increase in EXTEM clotting time. Platelet count slightly increased during the transplantation procedure, even in those patients without platelet concentrate transfusion. Maximum clot firmness, a resultant of fibrinogen concentration and platelet count and thus serving as an integral variable, remained almost constant. No patient left the operation theatre with clinically evident diffuse bleeding, no rescue medication (e.g. recombinant activated factor VIIa) was used.

**Table 3:** Haemostasis at the beginning and the end of liver transplantation as judged by conventional laboratory examinations and ROTEM™

	Initial values			Final values			
	Median	Percentiles		Median	Percentiles		Significance niveau
		25%	75%		25%	75%	
EXTEM							
CT (s)	55	46	78	62	51	83	*p* < 0.001
CFT (s)	119	82	177.5	163	117.5	251	*p* < 0.001
MCF (mm)	53	45	62	49	43	57	*p* < 0.001
Alpha (°)	68	59	75	64	54	72	*p* < 0.001
INTEM							
CT (s)	188.5	162	217.8	254.5	205	326.8	*p* < 0.001
CFT (s)	104	67	156	153.5	100	240.3	*p* < 0.001
MCF (mm)	52	45	61	49	42	56	*p* < 0.001
Alpha (°)	70	61	77	65	55	72	*p* < 0.001
FIBTEM							
CT (s)	54	45	75.5	61.5	50	83	*p* = 0.02
CFT (s)	*	*	*	*	*	*	
MCF (mm)	15	11	23	11	8	15	*p* < 0.001
alpha (°)	67	54.5	74	63	51	73	*p* < 0.001
APTEM							
CT (s)	64	51	88.5	71	55	93	*p* < 0.001
CFT (s)	128	92	182	167	119.5	247.5	*p* < 0.001
MCF (mm)	52	45.8	61	49	43	55	*p* < 0.001
alpha (°)	67	59	73	63	54	71	*p* < 0.001
INR	1.48	1.26	1.81	2.47	1.85	3.26	*p* < 0.001
aPTT (s)	45	36.9	56	81	58.1	108.9	*p* < 0.001
fibrinogen (mg/dl)	175.3	114.8	257	118	91	162	*p* < 0.001
antithrombin (%)	45.6	32	68.4	24.5	12.5	37.9	*p* < 0.001
platelets (1/10^3^ μl)	92	62	148.5	98	71	139	n.s.
Hb (g/dl)	10.1	8.9	11.5	9.9	9	11.1	*p* < 0.001

ROTEM™ values shown are clotting time (CT), clot formation time (CFT), maximum clot firmness (MCF), and angle alpha (alpha). Whole blood samples were activated with tissue factor (EXTEM™), elagic acid (INTEM™), tissue factor and platelet inhibition (FIBTEM™), and tissue factor and fibrinolysis inhibition (APTEM™). Conventional laboratory variables are international normalized ratio (INR), activated partial thromboplastin time (aPTT), fibrinogen concentration, antithrombin activity, platelet count, and hemoglobin concentration (Hb). ROTEM™ guided haemostasis management resulted in a slight decrease in most haemostatic variables during the transplantation procedure. Note, that CFT values are not generated in the FIBTEM assay. Differences between initial and final values were determined using the paired Wilcoxon test.

*: no clot formation detectable

## Discussion

Traditionally, fresh frozen plasma is used for correction of coagulopathy in liver transplantation procedures. However, increasing evidence questions the efficacy and suggests important risks of fresh frozen plasma. In view of these findings, the present study is the first to demonstrate that liver transplantations can be safely performed without fresh frozen plasma using a ROTEM™-guided substitution of coagulation factor concentrates.

### Haemostasis in patients presenting for liver transplantation

Haemostasis in patients presenting for liver transplantation varies with the underlying disease. In many patients, haemostasis is characterized by end stage liver failure with reduced synthesis capacity of coagulation factors as well as anticoagulant and fibrinolysis pathway proteins. The result is a rebalanced status prone to both bleeding and thrombosis [1, 15]. Thus, interventions in haemostasis in this very special surgical setting may potentially increase mortality by either insufficient treatment of diffuse bleeding or overtreatment. Indeed, there are many haemostatic derangements, which can occur during liver transplantation. Besides dilutional coagulopathy, trauma induced coagulopathy, aggravation of preexisting hypofibrinogenemia and a decrease of other coagulation factors due to consumption, thrombocytopenia as well hyperfibrinolysis and endogenous heparin-effects can result in diffuse bleeding [1]. Moreover, pulmonary embolism is, though rare, but a typical complication during the liver transplantation procedure [16].

It is important to note, that the decrease of coagulation factors is paralleled by the decrease of both anticoagulant and fibrinolytic proteins [17]. Therefore, a cautious substitution of coagulation factors avoiding overcorrection is highly recommended, and to achieve this goal, monitoring of haemostasis is mandatory.

### Monitoring of haemostasis during liver transplantation

Conventional laboratory tests are capable to diagnose a wide spectrum of haemostatic diseases. However, concerns arise from their long turnaround times [3]. Despite all efforts, turnaround times of laboratory assays often exceed 1 h due to transportation, centrifugation of samples, and the setup of the assay, and thus the delay for the initiation of the appropriate therapy is most often too long. Diffuse bleeding of any reason, which is not rapidly treated, can lead to dilutional coagulopathy and further worsening of blood loss.

Viscoelastic point-of-care coagulation assays like ROTEM™ can overcome several limitations of standard laboratory methods. The turnaround time of ROTEM™ analyses is considerably shorter and the combination of up to four parallel assays allows for a comprehensive characterization of the coagulation status [13]. The clotting time (CT) in tissue factor (EXTEM™) and elagic acid (INTEM™) activated ROTEM™ assays may serve as a measure for INR and aPTT, respectively (16). Thus, ROTEM™ enables the fast detection of deficiencies in coagulation factors from the extrinsic and intrinsic pathway, respectively, within approximately 5 min from blood sampling. Furthermore, clot firmness values obtained at 5 min after initial clotting are highly correlated with the maximum clot firmness eventually achieved during measurements [18]. Using clot firmness values of parallel tissue factor activated assays with (FIBTEM™) and without (EXTEM™) inhibition of platelet function with cytochalasin D allow for detection of thrombocytopenia and hypofibrinogenaemia [19]. Furthermore, heparin-effects (endogenous and exogenous) can be estimated by comparing the clotting times in the absence and presence of heparinase (INTEM™, HEPTEM™). Moreover, hyperfibrinolysis can be detected by the time course of clot firmness and low clot firmness values are associated with hyperfibrinolysis [20]. In this regard, it is important to state, that the diagnosis of minor to moderate hyperfibrinolysis can be made only after 30 to 60 min [21]. However, there is no standard laboratory assay available, which is capable of detecting hyperfibrinolysis at all, and minor or moderate hyperfibrinolysis often occurring after reperfusion is often self-limiting and not treated [8].

### Comparison of fresh frozen plasma based and coagulation factor based regimens

Fresh frozen plasma contains both pro- and anticoagulant factors as well as pro- and antifibrinolytic proteins. Many liver transplantation centres use a fresh frozen plasma based regimen, but the evidence for its prophylactic or therapeutic value is low [10]. In contrast, many issues regarding the safety and efficacy of fresh frozen plasma have been reported [9]. Risks associated with fresh frozen plasma transfusion are transmissions of infectious diseases, transfusion associated lung injury, and transfusion associated cardiocirculatory overload. Furthermore, large volumes of fresh frozen plasma transfused in the intention to improve haemostasis are likely to trigger additional RBC transfusions, as demonstrated in cardiac surgery [21].

Treatment of haemostatic disturbances with coagulation factors has, in theory, some advantages in comparison to fresh frozen plasma. Coagulation factors can be given according to a specific demand. They can be virus inactivated and are readily available as they are dissolved within a minute. Even huge amounts can be administered without the risk of volume overload. In general, fibrinogen, the most used coagulation factor in our setting, was judged to have a favourite safety profile in a recent meta-analysis [22]. Moreover, a weak recommendation for the use of fibrinogen in bleeding patients was given [11].

Prospective, randomized data comparing the administration of fresh frozen plasma and coagulation factor concentrates guided by point-of-care analyses for the treatment of bleeding are rare. However, as demonstrated in a sub-analysis of severely injured patients with blunt trauma, ROTEM™-guided coagulation management, exclusively using coagulation factor concentrates, corrected coagulopathy and was associated with less allogeneic transfusions, less multi organ failure and sepsis when compared to another cohort receiving additional fresh frozen plasma [23]. A first prospective, randomized trial comparing fresh frozen plasma to coagulation factor concentrates for the reversal of trauma induced coagulopathy has recently been terminated following an interim analysis indicating a possible harm for the patients randomized to the fresh frozen plasma treatment arm [24]. The authors concluded that treatment with coagulation factor concentrates is superior to the treatment with fresh frozen plasma.

### Bed side monitoring for the substitution of coagulation factors

Concerning the substitution of coagulation factors in liver transplantation, an adequate dosing can be assumed to be of outstanding importance to avoid overtreatment and thrombotic complication. For this purpose, a ROTEM™ based algorithm was used in the present study. It is an interesting finding, that many patients were not substituted with fibrinogen, PCC, platelet concentrates, and tranexamic acid at all. Most common was the substitution with fibrinogen concentrates (50.2% of cases), followed by tranexamic acid (24.5%), platelet concentrates (21.2%), and PCC (18.8%). This finding is in accordance with the fact, that fibrinogen is the coagulation factor to decrease first in bleeding patients [25]. In univariate analyses, all blood products as well as blood loss were associated with mortality, but the association was weak. Tranexamic acid was not associated with mortality in univariate analyses, which is in accordance to a recent meta-analysis in a recent retrospective analysis [26]. The examination of mortality in relation to the fibrinogen dose revealed, that fibrinogen concentrate did not increase mortality up to 8 g. Higher doses were rarely applied and the increased mortality in these patients was not related to haemostasis (with the possible exception of one patient with myocardial infarction). The safety of coagulation factor concentrates in the present study is further supported by multivariate analyses demonstrating that platelet concentrates, blood loss, and MELD score but not fibrinogen and PCC are independent predictors of mortality.

Keeping in mind, that substitution of coagulation factors was guided by ROTEM™, it becomes plausible, that the mortality is not related to fibrinogen concentrates and PCC. Accordingly, all bed side and laboratory coagulation values either slightly decreased or remained constant, demonstrating that a targeted correction of haemostasis can be achieved by repeated ROTEM™ analyses. Efficacy of the targeted treatment of haemostatic disturbances with coagulation factors without plasma transfusion is confirmed by the facts, that no patient left the operation theatre with clinically evident diffuse bleeding and that no rescue medication (e.g. recombinant activated factor seven) had to be applied.

### Limitations of the study

Of course, mortality in liver transplantation is dependent on numerous factors, namely the underlying liver disease and its severity, other diseases of the patients, quality of the graft as as well as surgical, anesthesiological and intensive care management. It is therefore obvious, that haemostasis management is only one out of several variables, which might affect outcome. In view of these uncertainties it is an important finding, that platelet transfusion is an important predictor of nonsurvival in our study as well as other studies while fibrinogen and PPSB had no effect on the outcome in multivariate analysis. The present study is the first to report retrospective mortality data using a ROTEM algorithm guided substitution of fibrinogen and PPSB for liver transplantations.

## Conclusions

The present study describes the ROTEM™ guided use of coagulation factor concentrates, platelet concentrates, and tranexamic acid for the management of haemostasis during liver transplantation without any use of fresh frozen plasma. The restrictive use of coagulation factor concentrates and tranexamic acid did not affect outcome, while platelet transfusion was associated with nonsurvival.

## Data Availability

The datasets used and/or analysed during the current study are available from the corresponding author on reasonable request.

## Abbreviations

FFP: Fresh frozen plasma
OR: Odds ratio
PCC: Prothrombin complex concentrate
